# Cerebrospinal fluid growth-associated protein 43 in multiple sclerosis

**DOI:** 10.1038/s41598-019-54032-1

**Published:** 2019-11-21

**Authors:** Åsa Sandelius, Sofia Sandgren, Markus Axelsson, Clas Malmeström, Lenka Novakova, Vesna Kostanjevecki, Manu Vandijck, Kaj Blennow, Henrik Zetterberg, Jan Lycke

**Affiliations:** 1 Department of Psychiatry and Neurochemistry, Institute of Neuroscience and Physiology at Sahlgrenska Academy, University of Gothenburg, Mölndal, Sweden; 2 Department of Clinical Neuroscience and Rehabilitation, Institute of Neuroscience and Physiology at Sahlgrenska Academy, University of Gothenburg, Gothenburg, Sweden; 3 Fujirebio Europe NV, Ghent, Belgium; 4 Clinical Neurochemistry Laboratory, Sahlgrenska University Hospital, Mölndal, Sweden; 5 Department of Neurodegenerative Disease, UCL Institute of Neurology, London, United Kingdom; 6 UK Dementia Research Institute at UCL, London, United Kingdom

**Keywords:** Diagnostic markers, Multiple sclerosis

## Abstract

Neurodegeneration in multiple sclerosis (MS) correlates with disease progression and reparative processes may be triggered. Growth-associated protein 43 (GAP-43) exhibits induced expression during axonal growth and reduced expression during MS progression. We aimed to evaluate if GAP-43 can serve as a biomarker of regeneration in relapsing-remitting MS (RRMS) and whether disease-modifying therapies (DMTs) influence GAP-43 concentration in cerebrospinal fluid (CSF). GAP-43 was measured using an enzyme-linked immunosorbent assay in 105 MS patients (73 RRMS, 12 primary progressive MS, 20 secondary progressive MS) and 23 healthy controls (HCs). In 35 of the patients, lumbar puncture, clinical assessment, and magnetic resonance imaging was performed before initiation of therapeutic intervention, and at follow-up. CSF GAP-43 concentration was significantly lower in progressive MS compared with HCs (*p* = 0.004) and RRMS (*p* =  < 0.001) and correlated negatively with disability (*p* = 0.026). However, DMTs did not alter CSF GAP-43. Interestingly, in RRMS CSF GAP-43 levels were higher in patients with signs of active inflammatory disease than in patients in remission (*p* = 0.042). According to CSF GAP-43 concentrations, regeneration seems reduced in progressive MS, increased during disease activity in RRMS but is unaffected by treatment of highly active DMTs.

## Introduction

Multiple sclerosis (MS) is an autoimmune disease of the central nervous system (CNS) that exhibits neurodegenerative features. The immune attack causes multiple demyelinating lesions with axonal loss but, with time, degeneration takes over with astrogliosis and atrophy^1,2^. However, regenerative mechanisms promote the repair of tissue damage, including remyelination in order to restore conduction. These pathogenic processes may be reflected by alterations in biomarker concentrations in cerebrospinal fluid (CSF), such as (i) neurofilament light (NFL), a biomarker of axonal damage^3–5^, (ii) glial fibrillary acidic protein (GFAP), a biomarker of astrogliosis^6^, and (iii) neurogranin, a marker of synaptic integrity^7^.

Growth-associated protein 43 (GAP-43), also known as B-50 or neuromodulin, is a membrane-associated protein^8^ and a major component of the motile growth cones of elongating axons and immature synaptic terminals^9^. GAP-43 is widely used as a marker of neuronal growth and regeneration, as it is highly expressed during synaptogenesis and axonal outgrowth^10,11^. Upon axotomy and in experimental models of ischemia, traumatic brain injury, and MS, GAP-43 protein expression is temporarily induced adjacent to the lesions^12–18^. Altered GAP-43 expression has also been reported in MS brains post-mortem^19,20^, including decreased expression in the vicinity of white matter lesions and increased or unaltered expression adjacent to remyelinated lesions^20^. CSF GAP-43 concentration correlates negatively with the Expanded Disability Status Scale (EDSS)^20^, and lower levels of CSF GAP-43 have been found in secondary progressive MS (SPMS) compared to early stages of MS, controls, and other neurological diseases^21^. Using a novel enzyme-linked immunosorbent assay (ELISA), we recently measured CSF GAP-43 in clinically isolated syndrome, early MS patients, and controls; though no differences were found between these major groups, patients who progressed had lower CSF GAP-43 concentrations^22^. In the present study, we investigated whether disease-modifying therapies (DMTs) alter CSF GAP-43 concentrations in MS, which would suggest an impact on regeneration.

## Results

### CSF GAP-43 concentrations in MS patients and healthy controls

**Figure 1:**
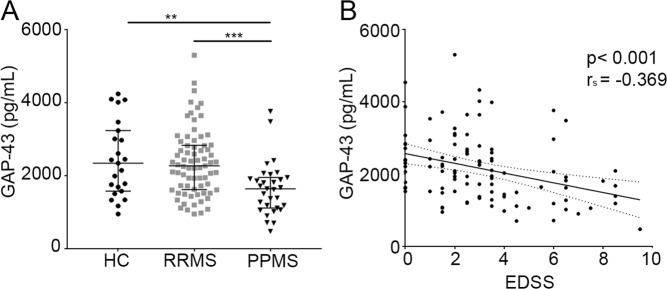
CSF GAP-43 differs across disease groups and correlates with EDSS: (**A**) CSF GAP-43 concentrations across disease groups in the MS population and HCs. **p = 0.0054, ***p < 0.0004. (**B**) Correlation between CSF GAP-43 concentration and EDSS in MS patients, *p* < 0.001. CSF, cerebrospinal fluid; GAP-43, growth-associated protein 43; MS, multiple sclerosis; HCs, healthy controls; RRMS, relapsing-remitting multiple sclerosis; PPMS, primary progressive multiple sclerosis; EDSS, Expanded Disability Status Scale.

Significantly lower CSF GAP-43 concentrations were found in progressive MS [1640 (1120–1950) pg/mL, *p* = 0.004], but not in relapsing-remitting MS (RRMS) [2270 (1620–2830) pg/mL, *p* = 0.8], compared with healthy controls (HCs) [2340 (1580–3230) pg/mL] (Fig. 1A). The difference was still significant when dividing the progressive MS patients into primary progressive MS (PPMS) and SPMS compared with HCs (each *p* =  < 0.05), and *p* = 0.003, and *p* = 0.002, respectively compared with RRMS. The diagnostic accuracy of CSF GAP-43 in diagnosing progressive MS, calculated with a ROC curve, gave an area under the curve (AUC) of 0.73 (*p* = 0.012).

### The influence of clinical and demographic factors and blood-brain barrier function on CSF GAP-43 concentrations

While no correlation was found between CSF GAP-43 concentrations and age in HCs, it correlated negatively with age (r_s_ = −0.339, *p* < 0.001), disease duration (r_s_ = −0.303, *p* = 0.002), and EDSS (r_s_ = −0.369, *p* < 0.001, Fig. 1B) in the MS population. Multiple regression analysis showed that only EDSS independently correlated with CSF GAP-43 concentration (*p* = 0.026). When dividing the MS population into RRMS, PPMS and SPMS patients, EDSS correlation was significant only in PPMS (PPMS: r_s_ = −0.651, *p* = 0.03, RRMS: r_s_ = −0.123, *p* = 0.301, SPMS: r_s_ = −0.242, *p* = 0.304). After adjustment for age in the PPMS group the correlation was still significant (*p* = 0.009). No significant differences were found between CSF GAP-43 concentrations in females and males, and baseline GAP-43 concentration did not correlate with the albumin ratio.

### CSF GAP-43 and disease activity

**Figure 2:**
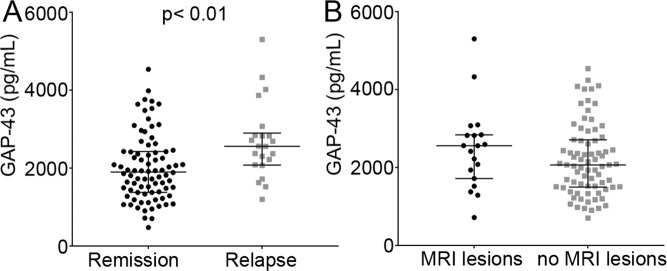
CSF GAP-43 and clinical characteristics: (**A**) CSF GAP-43 concentrations in RRMS patients with a relapse within 3 months prior to CSF sampling compared to CSF obtained in remission, *p* < 0.01. (**B**) CSF GAP-43 concentrations at baseline in MS patients with gadolinum-enhancing MRI lesions compared to those without lesions, *p* = 0.088. CSF, cerebrospinal fluid; GAP-43, growth-associated protein 43; RRMS, relapsing-remitting multiple sclerosis; MS, multiple sclerosis; MRI, magnetic resonance imaging.

CSF GAP-43 concentration was higher in RRMS patients with a relapse within 3 months prior to CSF sampling [2560 (2080–2900) pg/mL], compared with CSF obtained in remission [1900 (1380–2420) pg/mL, *p* = 0.002, Fig. 2A]. The RRMS patients with a relapse within 3 months were significantly younger than those without relapse (*p* = 0.011), and the latter had a longer disease duration (*p* < 0.001, RRMS, relapse mean = 2.3 years, no relapse mean = 10 years), after adjusted analysis for age and disease duration, disease duration was still significant (*p* = 0.02). Although, CSF GAP-43 concentrations at baseline were higher in patients with gadolinum-enhancing magnetic resonance imaging (MRI) lesions [2560 (1720–2840) pg/mL] compared with those without lesions [2030 (1470–2470) pg/mL], this difference was not statistically significant (*p* = 0.088, Fig. 2B).

### Effect of treatment on CSF GAP-43 concentration

**Figure 3:**
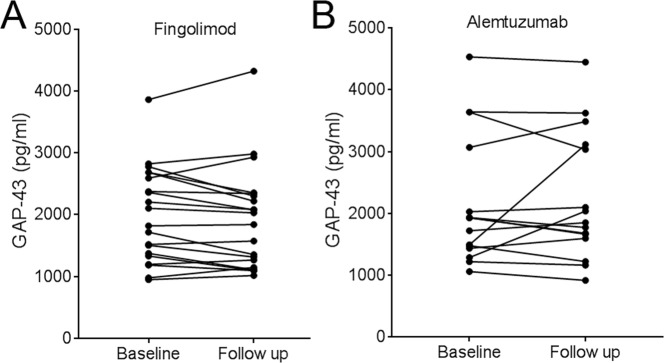
CSF GAP-43 at baseline and after DMTs: CSF GAP-43 concentrations at baseline and follow-up after fingolimod (**A**) and alemtuzumab (**B**) treatment. CSF, cerebrospinal fluid; GAP-43, growth-associated protein 43; DMTs, disease-modifying therapies.

At baseline, CSF GAP-43 concentrations were not different in patients on DMTs (*n* = 44) and treatment-naïve patients (*n* = 61, *p* = 0.851), or in patients on first line compared with second line treatment (*p* = 0.935). In patients switching from treatment-naïve (*n* = 1), first-line (*n* = 15) or second-line (*n* = 19) to fingolimod (*n* = 20) or alemtuzumab (*n* = 15), CSF GAP-43 concentrations did not change and the follow-up CSF GAP-43 level [fingolimod: 1940 (1180–2330) pg/mL, alemtuzumab: 1850 (1600–3120) pg/mL] was highly dependent on baseline concentrations [fingolimod: 1960 (1340–2660) pg/mL, alemtuzumab: 1930 (1440–3070) pg/mL, fingolimod: r_s_ = 0.958, *p* < 0.001, alemtuzumab: r_s_ = 0.793, *p* < 0.001] (Fig. 3A,B), with a mean coefficient of variance (CV) between these time points of 7.8% and 11.6%, respectively. At baseline, no significant difference in CSF GAP-43 concentrations were found between RRMS patients who achieved no evidence of disease activity (NEDA-3) (*n* = 19) switching from treatment naïve (*n* = 1), first-line (*n* = 15) or second-line (*n* = 19) to fingolimod (*n* = 20) or alemtuzumab (*n* = 15), and those who did not [NEDA-3: 1669 (1298–2244) pg/mL, no NEDA-3: 2018 (1482–2788) pg/mL, *p* = 0.26]. At follow-up, we neither found a significant difference in CSF GAP-43 concentrations between patients with NEDA-3 [*n* = 23, 1619 (1322–2244) pg/mL] and those with no NEDA-3 [2201 (1569–2754) pg/mL, *p* = 0.160]. In the group of RRMS patients that switched to fingolimod or alemtuzumab there was a slight trend that those with higher baseline CSF GAP-43 concentration more often achieved clinical disability improvement (CDI; EDSS improvement of ≥1 point for those with an EDSS of ≥ 2 points at baseline, excluding 5 patients as they did not have EDSS ≥ 2 at baseline), but not significant [CDI at follow-up (*n* = 5): 1930 (1471–2999) pg/mL, no CDI at follow-up (*n* = 25): 1717 (1354–2398) pg/mL, *p* = 0.58]. Examining treatment effects on CSF GAP-43 concentrations in strata of patients who either started on first-line treatment and changed to second-line treatment or changed from one type of second-line treatment to another second-line treatment, did not reveal any significant changes in CSF GAP-43 concentration (*p* = 0.143 and *p* = 0.54, respectively).

## Discussion

The data in this study are based on a heterogeneous group of MS patients in different stages of disease. We confirmed that CSF GAP-43 was significantly lower in progressive MS compared with HCs and RRMS patients^21,22^, with the lowest levels in PPMS. CSF GAP-43 concentrations correlated negatively with age, disease duration, and EDSS, but only independently with EDSS. However, we found that the CSF GAP-43 concentration was significantly higher in RRMS patients with signs of active inflammatory disease compared with RRMS patients in remission, whereas fingolimod and alemtuzumab treatment did not alter CSF GAP-43 concentrations.

We confirmed no difference between CSF GAP-43 concentrations of RRMS and HCs^22^. In contrast, the GAP-43 was reduced in progressive MS, suggesting lost or reduced regenerative potential in late MS. This ability seems to become more marked with increased disability and may be the result of atrophy development and progressive neuro-axonal loss. This finding is in contrast to our findings in Alzheimer’s disease, in which CSF GAP-43 was increased^23^. Thus, the nature of neurodegeneration seems to be more important than the degree of neurodegeneration, as the level of CSF GAP-43 was not increased in other neurodegenerative diseases^23^ and not related to the extent of atrophy^24^. Although the pathogenesis of MS progression is unknown, and clearly different from that of Alzheimer’s disease, our results suggest that regenerative processes such as synaptogenesis and axonal outgrowth, is reduced in progressive MS. This interpretation is supported by the decreased expression of GAP-43 in the vicinity of white matter lesions^20^.

Our results suggest that the CSF GAP-43 concentration increases in association with new inflammatory activity in MS patients. Increased CSF GAP-43 concentration was found in RRMS and patients with clinically isolated syndrome with >10 T2 lesions compared with those with fewer T2 lesions^22^. However, increase of CSF GAP-43 during relapses or in the presence of contrast enhancing lesions has not been reported before. This elevation seemed independent of blood-brain barrier function, since no correlation was found between CSF GAP-43 and the albumin ratio. Previous studies of axotomy and experimental models of ischemia, traumatic brain injury, and MS show that GAP-43 protein expression is induced temporarily and adjacent to neuro-axonal damage and the formation of new lesions^12–18^. Thus, immune-mediated damage of the CNS may explain the transient release of GAP-43 that we found in the CSF of MS patients with ongoing disease activity. Another explanation could be that the CSF GAP-43 concentration increases during MS relapse in an attempt to regenerate injured axons.

We could not show any significant impact of DMTs on CSF GAP-43 concentration. Similar CSF GAP-43 concentrations were observed at baseline in patients without prior treatment and those on first- or second-line treatment. MS treatments primarily reduce CNS inflammation in MS, and not the regenerative capacity. The lack of change in CSF GAP-43 across different therapies suggests that reduced inflammation does not influence regeneration involving GAP-43.

HCs were of younger age than the MS population. However, we found no association between CSF GAP-43 concentrations and age in HCs. While multiple regression analysis revealed an independent relationship between disability and the CSF GAP-43 concentration, this were not the case for disease duration and age. Thus, our study confirmed previous findings^22,23,25^, and thus, differences in age between HCs and patients should not have influenced the results. Similar differences existed between the gender distributions of the study groups. However, neither did gender seem to influence the CSF GAP-43 concentrations. Moreover, we could only report an association between ongoing inflammatory activity and increased CSF GAP-43 levels, but we lacked MRI data on lesion load or cerebral and spinal cord atrophy. Relationships between CSF GAP-43 concentrations and such MRI measures should be further explored to better characterize the possible role of GAP-43 in the pathogenesis of MS.

In conclusion, studies of GAP-43 in MS concordantly show that, this protein is decreased in CSF in progressive MS, and we found an association with disability and also with disease activity. However, effective DMTs had no effect on the CSF GAP-43 concentration. Previous studies have not shown correlation between GAP-43 and NFL in CSF^22^, indicating that axonal damage does not influence the release of GAP-43 in CSF. Although the clinical potential of GAP-43 as a biomarker in MS seems limited at this stage, it contributes to further understand the pathogenesis behind progression, and that of degeneration and regeneration in MS.

## Methods

### Patients and healthy control subjects

We included 23 HCs and 105 MS patients, including 73 with RRMS, 20 with SPMS, and 12 PPMS, fulfilling the revised McDonald criteria from 2010^26^. Ninety of these patients had previously participated in studies of CSF biomarkers in MS^6,27^, including one investigating the influence of fingolimod treatment^28^. The remaining patients (*n* = 15) were recently recruited from consecutive patients at the MS Centre, Sahlgrenska University Hospital, Gothenburg, Sweden, to explore the influence of alemtuzumab therapy on CSF biomarker concentrations. At baseline, 24 patients received first-line treatment (19 interferon beta, 4 glatiramer acetate, 1 dimethyl fumarate), 20 received second-line treatment (6 fingolimod, 1 rituximab, 13 natalizumab) and 61 were treatment naïve. Descriptive clinical and demographic characteristics are presented in Table 1.

**Table 1:** Descriptive clinical and demographic characteristics of patients and HCs.

N	HCs	RRMS	PPMS	SPMS
	23	73	12	20
Mean age, years (SD)	29.3 (10.1)	39.2 (10.3)^a^	52.4 (7.0)^b^	53.3 (8.7)^b^
Gender, female/male	14/9	48/25^c^	6/6	7/13
Disease duration, years	NA	6 (2–12.5)	5.5 (1.25–8)	18 (12.3–22.8)^d,e^
EDSS	NA	2 (1–3)	4.5 (3–6)^f^	6.5 (5.1–8.4)^g^
Relapse 3 months prior to LP, yes/no	NA	22/51	0/12	0/20
**DMT**				
No previous treatment	NA	34	11	16
First-line treatment	NA	21	0	3
Second-line treatment	NA	18	1	1
QAlb	3.9 (3.3–5.8)	4.9 (3.9–6.32)	5.2 (4.4–6.0)	6.9 (5.1–9.7)
	**Fingolimod**		**Alemtuzumab**	
N	20		15	
Mean age, years (SD)	38.5 (10.3)		40.3 (7.7)	
Gender, female/male	9/11		9/6	
Disease duration, years	7 (3–11.8)		4 (3–13)	
EDSS	3 (1.1–3.5)		2.5 (1.5–3.5)	
Relapse 3 months prior to LP, yes/no	6/14		0/15	
**DMT baseline**				
No previous treatment	1		0	
First-line treatment	13		2	
Second-line treatment	6		13	
Q_Alb_	5.1 (3.2–6.6)		5.2 (3.2–6.4)	

Data are presented as n or median (interquartile range) unless otherwise noted. HCs, healthy controls; RRMS, relapsing-remitting multiple sclerosis; PPMS, primary progressive multiple sclerosis; SPMS, secondary progressive multiple sclerosis; EDSS, Expanded Disability Status Scale; LP, lumbar puncture; DMT, disease-modifying therapies; Q_Alb_, albumin ratio. First-line treatment = interferon beta, glatiramer acetate, dimethyl fumarate. Second-line treatment = natalizumab, fingolimod, rituximab.

^a^*p* < 0.05 RRMS versus HCs.

^b^*p* = 0.001 PPMS or SPMS versus HCs.

^c^*p* < 0.01 female versus male in RRMS.

^d^*p* < 0.001 SPMS versus RRMS.

^e^*p* < 0.01 SPMS versus PPMS.

^f^*p* < 0.05 PPMS versus RRMS.

^g^*p* < 0.001 SPMS versus RRMS.

### Clinical evaluation, sampling of CSF, and magnetic resonance imaging

All patients were assessed clinically at baseline by MS-specialized neurologists. The EDSS^29^ was used to score neurological deficits and impairment. A relapse was defined as an episode of neurological disturbance lasting for at least 24 h that could not be better explained by another cause^30^. Lumbar puncture was performed at baseline (*n* = 127), and in the fingolimod (*n* = 20) and alemtuzumab (*n* = 15) treatment groups, CSF was obtained again after a median of 7 (range 3–13) and 24 (range 24–26) months, respectively. One patient had only a follow-up lumbar puncture sample. The CSF samples were handled according to the consensus protocol of the BioMS-EU network for CSF biomarker research in MS^31^. MRI of the brain was performed on 66 patients at baseline and in close association with the clinical neurological examinations and lumbar puncture (median 1 month, range 0–7 months). A standard MRI protocol for MS including intravenous gadolinium contrast was performed on a 1.5 or 3 Tesla MRI scanner and included T1, T2, and fluid attenuation inversion recovery (FLAIR) sequences, according to the Swedish guidelines^32^.

### CSF GAP-43 analysis

The GAP-43 protein concentration in CSF was determined by an in-house ELISA as described previously^22^, with minor modifications. Briefly, plates were coated with NM4 monoclonal antibody (1.35 μg/mL, Fujirebio, Tokyo, Japan) in carbonate buffer (pH 9.6), and incubated over night at 4 °C. After three washes with phosphate-buffered saline with tween (PBST), wells were blocked with a solution of PBST-milk (2% non-fat dry milk, Biorad, Hercules, CA, US) for 1 hour on a shaker at room temperature and placed at −20 °C for at least 12 hours to enable higher throughput during sample runs. After thawing and three more washes, the detection antibody (polyclonal ABB-135, Nordic Biosite, Täby, Sweden), 50 μL of twofold prediluted samples, and calibrators (recombinant GAP-43) in PBST-milk were co-incubated overnight at 4 °C. Plates were washed three times and secondary antibody (anti-rabbit IgG HRP, Promega, Wisconsin, US) diluted in 1% bovine serum albumin (BSA)/PBST at 1:20000 added and incubated on the bench for 2 hours at room temperature. Wells were washed and 100 μL of 3,3′,5,5′-tetramethyl-benzidine (TMB One, KemEnTech Diagnostics, Taastrup, Denmark) added. Plates were incubated in the dark for 30 min before adding 100 μL of 0.2 M H_2_SO_4_ and measuring the absorbance at 450 nm immediately, with a 650 nm reference, on a SunriseTM microplate absorbance reader (Tecan group, Männedorf, Switzerland). The analysis was carried out using the same batch of reagents and the analyst was blind to disease condition. Quality control samples were run for estimation of intra- and inter-plate variations. The intra-assay CV for a sample with a mean concentration of 3597 pg/mL was 5.4% with an inter-assay CV of 8.9%, and for sample with a mean concentration of 571 pg/mL the intra- and inter-assay CVs were 10.7% and 11.1% respectively. The lower limit of quantification (LLoQ), determined as the lowest concentration at which GAP-43 could be detected reliably, was 475 pg/mL after adjusting for a twofold sample dilution. Further assay evaluation of precision, dilution linearity, spike recovery in matrix, and sample stability has been described previously^25^.

### Albumin ratio

Serum and CSF albumin concentrations were measured by immunonephelometry on a Beckman Immage Immunochemistry system (Beckman Instruments, Beckman Coulter, Brea, CA, USA). Q_Alb_ was calculated as the ratio of CSF albumin (mg/L) to serum albumin (g/L)^33^.

### Statistical analysis

Data were not normally distributed; therefore, non-parametric tests were used. Differences across patient groups, clinical measures, and treatments were evaluated as continuous variables using Kruskal-Wallis or Mann-Whitney U tests. The Wilcoxon signed ranks test for paired samples was used to evaluate changes in CSF GAP-43 over time in patients switching from other treatments to fingolimod or alemtuzumab. Possible correlations between biomarker concentrations and clinical measures were evaluated using Spearman correlation. The chi-squared test was used for categorical variables. Multiple regression analysis was performed to test the influence of age, disease duration, and EDSS on CSF GAP-43 concentration. SPSS version 23.00 (IBM, NY, US) and GraphPad Prism 5.0 (GraphPad Inc., California, USA) were used for statistical analyses. All tests were two-sided with a significance threshold of *p* < 0.05.

### Ethical standards

All patients and HCs voluntarily participated in the study, and informed consent was obtained from all subjects. Measures were taken to minimize pain and discomfort for all participants in the study, and all methods were performed in accordance with the relevant ethical guidelines and regulations. The study conforms with The Code of Ethics of the World Medical Association (Declaration of Helsinki)^34^. For patient material from previous studies, reference is made to the respective publication^6,27,28^ for their ethical approval. For the remaining patients and for HCs, participating in the assessment of alemtuzumab, the study was approved by the Regional Ethics Review Board in Gothenburg, Sweden (Reference number 460–13).

## Data Availability

The datasets generated and/or analyzed during the current study are available from the corresponding author on reasonable request.
